# What’s coming for health science and policy in 2018? Global experts look ahead in their field

**DOI:** 10.1371/journal.pmed.1002498

**Published:** 2018-01-30

**Authors:** The PLOS Medicine Editors, Soumya Swaminathan, Robin S. Room, Louise C. Ivers, Graham Hillis, Rebecca F. Grais, Zulfiqar A. Bhutta, Peter Byass

**Affiliations:** 1 Public Library of Science, San Francisco, California, United States of America, and Cambridge, United Kingdom; 2 World Health Organization, Geneva, Switzerland; 3 Centre for Alcohol Policy Research, La Trobe University, Bundoora, Victoria, Australia; 4 Centre for Social Research on Alcohol and Drugs, Stockholm University, Stockholm, Sweden; 5 Center for Global Health, Massachusetts General Hospital, Boston, Massachusetts, United States of America; 6 Department of Global Health and Social Medicine, Harvard Medical School, Boston, Massachusetts, United States of America; 7 Department of Cardiology, Royal Perth Hospital, Perth, Australia; 8 University of Western Australia, Crawley, Australia; 9 Epicentre, Paris, France; 10 Centre for Global Child Health, The Hospital for Sick Children, Toronto, Canada; 11 Centre of Excellence in Women and Child Health, Aga Khan University, Karachi, Pakistan; 12 Department of Public Health and Clinical Medicine, Umeå University, Umeå, Sweden; 13 University of Aberdeen, Aberdeen, Scotland, United Kingdom; 14 University of the Witwatersrand, Johannesburg, South Africa

## Abstract

In *PLOS Medicine*’s first editorial of 2018, editorial board members and other leading researchers share their hopes, pleas, concerns, and expectations for this year in health research and policy.

*PLOS Medicine*’s commitment to bringing research and commentary on urgent, grievous, and underaddressed health needs to an open-access platform has never felt more relevant than in 2017. A study [1] quantifying police activity-related deaths in the United States, published in October, received more than 100,000 views within a few weeks of publication, and our Special Issue on Advances in HIV Research led November to a journal record for number of articles published in a single month. Meanwhile, requirements for sharing data, which PLOS launched in 2014, expanded across medical publishers [2], indicating that globally important findings in health research, and the ability to further analyze these, will become increasingly accessible in coming years.

In 2018, we plan a Special Issue on cardiovascular disease in the context of comorbid conditions and another probing the interaction between climate change and health. What further priorities must research and policy address? For our first editorial of 2018, we asked editorial board members and other leading researchers to share their hopes, pleas, concerns, and expectations for medical research and policy related to global health in 2018.

## Tuberculosis research in 2018: Expect translation

### Soumya Swaminathan

The past year saw unprecedented political engagement on tuberculosis (TB). The Moscow Declaration to End TB is a promise to increase multisectoral action as well as track progress and build accountability. The first United Nations General Assembly High-Level Meeting on TB in 2018 will seek further commitments from heads of state, which some, like the Prime Minister of India, have already made.

However, TB still kills more people than any other infectious disease globally, and treatment of multidrug-resistant TB is a challenge. Seven countries account for 64% of the total incidence of TB, with India leading the count, followed by Indonesia, China, Philippines, Pakistan, Nigeria, and South Africa [3]. Major drivers of TB include poverty, overcrowding and undernutrition, and HIV in sub-Saharan Africa. Major gaps remain in the understanding of pathogenesis of some forms of TB, interaction between mycobacterial lineage and host genetics, determinants of virulence and transmission, biomarkers that can predict progression from latent to active disease, and correlates of protective immunity [4].

The coming year will likely see advances in a few areas. The establishment of international networks of cohorts of TB patients, their household contacts, and healthy controls (e.g., Regional Prospective Observational Research for Tuberculosis [RePORT] International) will provide the kind of specimen repositories that basic scientists and technology developers have dreamt about, leading to more productive translational research [5]. Better diagnostics, mainly from India and China, are on the horizon, making it possible that every patient with suspected TB could have a molecular diagnostic test with susceptibility testing to at least rifampicin, even at a remote primary health center, with no electricity or highly qualified staff. More data are likely to come on the combination of bedaquiline and delaminid in treating extensively drug-resistant TB.

Most exciting is the recognition that there are similarities in changes in the regulation of immune responses to malignancies and infectious pathogens that involve modulation of common signaling pathways [6]. Identifying these specific immune cell core regulatory pathway disruptions will be key to developing new host-directed therapies for treatment and prevention.

## For alcohol policy in 2018, the options are good, but expectations are limited

### Robin Room

On May 1, 2018, minimum unit pricing for alcohol comes to Scotland after a 6-year blockage by a tooth-and-nail legal counteroffensive by alcohol industry interests. In the 6 years before it expires if not renewed [7], the legislation is likely to have a moderate effect: one estimate is that the new minimum price, 50p per alcohol unit, will reduce annual alcohol-attributable deaths by 3.5% after 1 year and by double that if continued for 20 years [8].

What else can be expected in 2018 in terms of alcohol policies that would reduce alcohol-related harm? There is broad evidence concerning policies that are effective in reducing levels of consumption and of harm from drinking [9], but the likelihood of substantial policy changes affecting availability or promotion is small.

Part of the problem in adopting a coherent set of alcohol policies is the wide range of types of harm involved, for which diverse government departments are responsible. But perhaps the biggest stumbling block is the political power of alcohol industry interests in fighting them off. For example, in 2018, it will be interesting to watch what happens in Australia with a new National Alcohol Strategy for 2018 to 2026. A draft has now been circulated for comments, due by February 11, 2018. But the final strategy will need to look quite different if it is to be a real strategy with proposed actions related to goals and clear indications of how they will be decided and acted upon. Even at this stage, industry interests have moved to forestall an effective strategy through the media, highlighting the draft’s mention of minimum unit pricing as one among many possible options [10]. Asked about this, the federal health minister promptly disavowed the document [11]. Scotland’s principled and sustained action on alcohol policy sets a standard to aim for, but in Australia, as in many other places, it will be difficult to match.

## Infectious disease outbreaks in Yemen: A man-made disaster that has a solution

### Louise Ivers

One thousand days of war in Yemen have resulted in many thousands of civilian deaths as well as deliberate destruction of health infrastructure and water infrastructure and disruption in the payment of thousands of public servants [12]. Restrictions on imports have prevented access to food, medicine, and humanitarian supplies [13]. In 2017, almost 1 million cases of suspected cholera were reported in the country, and although laboratory infrastructure is lacking to confirm every single case, the sheer scale of the epidemic of watery diarrhea is staggering. Frontline workers have privately reported their fears that cholera is being deliberately used as a weapon of war by parties to the conflict. Publicly, despite pleas from health workers and UN agencies for humanitarian space to provide relief, blockades and bombings continue. Diphtheria cases are now also on the rise—a painful disease, with a high mortality rate that occurs when vaccination rates are too low.

These epidemics, as well as the dire lack of access to food (already 400,000 children with severe acute malnourishment), do not bode well for 2018 [14]. Even if the fighting stopped tomorrow, the impact of these health crises would be felt for a generation. The continued hostilities are like a genocidal experiment to demonstrate just how badly the destruction of a health system can destroy the health of a nation. Every one of these medical emergencies—cholera, diphtheria, famine—is completely preventable and treatable. Without an end to the conflict, or at a minimum an agreement to open corridors of reliable safe humanitarian access, the ordinary people of Yemen will continue to suffer despite the fact that the public health community has the knowledge and the tools to stop the epidemics and save lives.

## Changing paradigms for atherosclerosis

### Graham Hillis

In the past year, landmark studies have signaled important potential advances in our ability to treat and prevent atherosclerotic disease.

The Further Cardiovascular Outcomes Research with PCSK9 Inhibition in Subjects with Elevated Risk (FOURIER) trial confirmed that evolocumab, a humanized monoclonal antibody inhibitor of proprotein convertase subtilisin/kexin type 9 (PCSK9), resulted in a marked reduction in low-density lipoprotein cholesterol (LDL-C). [15] Importantly, it also demonstrated a modest but significant reduction in adverse cardiovascular outcomes even in well-treated patients followed up over a relatively short period [15]. Trials using bococizumab [16] and alirocumab [17] suggest comparable benefits from inhibition of PCSK9, and the results of the large Evaluation of Cardiovascular Outcomes After an Acute Coronary Syndrome During Treatment With Alirocumab (ODYSSEY Outcomes) trial testing this latter agent are anticipated in 2018 [18,19]. Further studies are also underway testing alternate methods influencing lipid pathways using small interfering RNAs and antisense oligonucleotides. LDL-C reduction with statins greatly improves the prognosis of patients with atherosclerotic disease, and this early evidence suggests that additional reductions using novel therapies will further reduce clinical events, but, inevitably, the incremental benefits from further lipid lowering may diminish. Potentially the most import recent development in the management of atherosclerotic disease has therefore been the publication of the Canakinumab Anti-inflammatory Thrombosis Outcomes Study (CANTOS) [20]. This demonstrated that treatment of patients with prior myocardial infarction and persistently elevated C-reactive protein with canakinumab, a monoclonal antibody that targets the proinflammatory cytokine interleukin 1 beta (IL-1β), reduces major cardiovascular events [20]. Again, the reduction was modest and driven by nonfatal events. It was, however, observed in patients who were otherwise well treated and was independent of lipid lowering. Thus, the CANTOS trial supports the hypothesis that atherosclerosis is an inflammatory disease and raises the prospect of treatments that target this (as yet largely unaddressed) pathogenic mechanism. Other such therapies are currently being tested and developed.

These novel treatments are promising but extremely expensive. The challenges will be to reduce these costs, identify those patients who may derive the most benefit, and develop safe and inexpensive alternatives. Only then are such therapies likely to result in a meaningful reduction in the global burden of atherosclerotic disease.

## Beyond words: US federal funding for public health and science

### Rebecca Grais

According to media reports, Centers for Disease Control and Prevention (CDC) officials prohibited the use of 7 words and phrases in developing budget requests [21]. CDC and US Department of Health and Human Services (HHS) officials responded to reports by stating that there was no ban or censorship [22,23]. Media coverage has centered on either condoning the “ban” as evidence of the administration’s commitment to reducing government spending or on denouncing it as yet another piece of evidence of the administration’s antiscience agenda.

There is no doubt that evidence-based policy solutions and protecting vulnerable populations have not been a priority of this administration, with or without a word ban. Budget cuts or simply maintaining current funding to the CDC, as well as to National Institutes of Health (NIH) and other science and public health agencies and initiatives, will be felt directly through the scale back of existing public health programs, research funding, and initiatives. They will be felt indirectly by reinforcing the message that certain populations and conditions are not of interest to the US government. Cuts or removal of some programs, such as the HHS Secretary’s Minority AIDS Initiative Fund (SMAIF) [24], which provides funding to reduce HIV-related health disparities in racial and ethnic minority communities, would have dire consequences for the most vulnerable. Irrespective of whether words are banned or simply avoided by CDC staff, the consequences of such cutbacks remain the same for vulnerable populations, whose words are often not heard at all.

## Next steps for a post-transition WHO

### Peter Byass

Since Dr. Tedros took over as WHO Director-General in mid-2017 [25], much has been discussed and written about WHO’s future [26,27]. Early 2018 marks the end of the beginning, as Dr. Tedros’ new leadership team finds its feet [28]. WHO has been widely criticized for the vertical silos in which many important issues reside. So now, with a number of discrete new priorities having been identified [29], the overarching issue is how WHO can move forward in an integrated way, forging essential links between important topics.

WHO’s central aim of promoting universal health coverage (UHC) is inextricably linked with caring for increasing numbers of people living with noncommunicable diseases (NCDs) as life expectancy increases and populations age, and UHC and NCD outcomes are critical components of the UN Sustainable Development Goals (SDGs). Effective UHC is similarly a prerequisite for improving reproductive and child health, targeted by other SDGs. Environmental, climate-change, and emergency challenges to health are further priorities closely linked with UHC, NCDs, and SDGs. Finally, progress across all these priorities must be measurable via much-improved data at the grassroots level, forming integrated metrics.

Thus, I hope that during 2018 WHO will move on from a shopping list of priorities to a web of interconnected tasks that can be collectively pursued at WHO’s Geneva headquarters, in Regional Offices and within all Member States.

## Global maternal and child health: Many gains, but much more remains

### Zulfiqar Bhutta

The Millennium Development Goals (MDGs) concluded at the end of 2015 and were replaced by the much anticipated SDGs [30]. While maternal and child health (MCH) enjoyed tremendous primacy during the MDG period, MCH advocates and other health experts have reason for concern: the 17 SDGs now include just 1 health-related goal (SDG3). However, the SDGs are intimately interconnected, and a range of other goals pertain to MCH, such as SDG2 for eliminating hunger, SDG5 for achieving gender equality, SDG6 for assuring clean water and sanitation, and many others related to sustainable and safe living environments, as well as those addressing climate change. SDG10 for reducing inequalities is especially pertinent. Notably, while many countries achieved major reductions in maternal and child mortality during the MDG period, others were left behind. In many instances, national averages masked tremendous disparities and differentials between geographic regions and among marginalized populations [31]. Additionally, relatively few interventions focused on going beyond survival to addressing human development [32].

Moving forward, initiatives to address maternal and child survival, health, and development outcomes within the SDG framework must consider opportunities for reaching hitherto marginalized populations and overcoming barriers that have constrained uniform progress. In many countries with a high burden of maternal and child mortality, these include conflict and humanitarian emergencies, accentuated by climate change [33]. In other instances, rapid urbanization and rising slum populations pose challenges but also provide opportunities for innovation and outreach [34]. Addressing these issues at an early stage of the SDGs would ensure that the vision of UHC can be realized for all in need, everywhere!

## Abbreviations

CANTOS: Canakinumab Anti-inflammatory Thrombosis Outcomes Study
CDC: Centers for Disease Control and Prevention
FOURIER: Further Cardiovascular Outcomes Research with PCSK9 Inhibition in Subjects with Elevated Risk
HHS: United States Department of Health and Human Services
IL-1β: interleukin 1 beta
LDL-C: low-density lipoprotein cholesterol
MCH: maternal and child health
MDG: Millennium Development Goal
NCD: noncommunicable disease
NIH: National Institutes of Health
PCSK9: proprotein convertase subtilisin/kexin type 9
RePORT: Regional Prospective Observational Research for Tuberculosis
SDG: Sustainable Development Goal
SMAIF: Secretary’s Minority AIDS Initiative Fund
TB: tuberculosis
UHC: universal health coverage
